# Hemodynamic comparison of different multisites and multipoint pacing strategies in cardiac resynchronization therapies

**DOI:** 10.1007/s10840-018-0362-y

**Published:** 2018-04-07

**Authors:** Francesco Zanon, Lina Marcantoni, Enrico Baracca, Gianni Pastore, Giuseppina Giau, Gianluca Rigatelli, Daniela Lanza, Claudio Picariello, Silvio Aggio, Sara Giatti, Marco Zuin, Loris Roncon, Domenico Pacetta, Franco Noventa, Frits W. Prinzen

**Affiliations:** 1 Arrhythmia and Electrophysiology Unit, Santa Maria Della Misericordia General Hospital, Rovigo, Italy; 2 Cardiology Department, Santa Maria Della Misericordia General Hospital, 140, Viale Tre Martiri, 45100 Rovigo, Italy; 3 Interventional Cardiology Unit, Santa Maria Della Misericordia General Hospital, Rovigo, Italy; 4 St. Jude Medical, Agrate Brianza, Italy; 5 Department of Molecular Medicine, University of Padua, Padua, Italy; 6 Cardiovascular Research Institute Maastricht (CARIM), Maastricht, The Netherlands

**Keywords:** Heart failure, Multisite pacing, Multipoint pacing, Dual LV site pacing, Hemodynamic optimization, Electrical delay

## Abstract

**Purpose:**

In order to increase the responder rate to CRT, stimulation of the left ventricular (LV) from multiple sites has been suggested as a promising alternative to standard biventricular pacing (BIV). The aim of the study was to compare, in a group of candidates for CRT, the effects of different pacing configurations—BIV, triple ventricular (TRIV) by means of two LV leads, multipoint (MPP), and multipoint plus a second LV lead (MPP + TRIV) pacing—on both hemodynamics and QRS duration.

**Methods:**

Fifteen patients (13 male) with permanent AF (mean age 76 ± 7 years; left ventricular ejection fraction 33 ± 7%; 7 with ischemic cardiomyopathy; mean QRS duration 178 ± 25 ms) were selected as candidates for CRT. Two LV leads were positioned in two different branches of the coronary sinus. Acute hemodynamic response was evaluated by means of a RADI pressure wire as the variation in LVdp/dtmax.

**Results:**

Per patient, 2.7 ± 0.7 veins and 5.2 ± 1.9 pacing sites were evaluated. From baseline values of 998 ± 186 mmHg/s, BIV, TRIV, MPP, and MPP-TRIV pacing increased LVdp/dtmax to 1200 ± 281 mmHg/s, 1226 ± 284 mmHg/s, 1274 ± 303 mmHg, and 1289 ± 298 mmHg, respectively (*p* < 0.001). Bonferroni post-hoc analysis showed significantly higher values during all pacing configurations in comparison with the baseline; moreover, higher values were recorded during MPP and MPP + TRIV than at the baseline or during BIV and also during MPP + TRIV than during TRIV. Mean QRS width decreased from 178 ± 25 ms at the baseline to 171 ± 21, 167 ± 20, 168 ± 20, and 164 ± 15 ms, during BIV, TRIV, MPP, and MPP-TRIV, respectively (*p* < 0.001).

**Conclusions:**

In patients with AF, the acute response to CRT improves as the size of the early activated LV region increases.

## Introduction

A selected group of patients with systolic heart failure (HF) and prolonged QRS may benefit from Cardiac Resynchronization Therapy (CRT) [1, 2]. However, one third of patients referred for this therapy do not show a favorable long-term outcome [3]. One of the reasons for the lack of response is the suboptimal position of the left ventricular (LV) pacing lead [4]. As described in our previous paper [5], we usually target the most electrically delayed site in order to achieve the optimal resynchronization. Novel techniques suggested by some authors are based on the concept that stimulating a larger area of the LV may improve the success rate of CRT. Multisite LV pacing can be carried out with several LV leads in separate coronary sinus (CS) veins [6, 7] or by means of a single multipolar lead capable of delivering multiple stimuli within one CS vein [8–11]. Our study aimed to compare the acute effects of four pacing configurations in a selected group of patients in permanent atrial fibrillation (AF): biventricular (BIV), multiple site pacing by means of two LV leads (triple ventricular, TRIV), multipoint pacing (MPP) through a single quadripolar lead, and the combination of TRIV and MPP.

## Methods

Fifteen consecutive patients with permanent AF and indications for the implantation of a CRT-device, in accordance with the European Society of Cardiology/European Heart Rhythm Association (ESC/EHRA) guidelines [12], were enrolled in a prospective study.

In our center, the dual LV site technique is part of common practice in AF patients who are candidates for CRT. We usually optimize the best pacing site during the CRT implantation procedure by means of Q-LV and hemodynamic measurements. The implanting method was described in our previous manuscript [13]. Briefly, the right ventricular (RV) lead was implanted in the mid-septum, according to our standard implantation procedure. Cannulation of the CS and sub-cannulation of all suitable collateral veins were performed by means of a telescopic approach.

Pacing sites were classified as anterior, antero-lateral, lateral, postero-lateral, and posterior segments (in the left anterior oblique view) and as basal, mid, and apical ventricular segments (in the right anterior oblique view), in accordance with the scheme established by Singh et al. [14].

Suitable pacing sites were systematically screened by measuring the local electrical delay (Q-LV) during intrinsic activation of the LV, by means of a BARD Labsystem Pro EP V2.4a (C.R. Bard Inc., Lowell, MA), and the LVdp/dtmax by means of a Certus Pressure Wire and PhysioMon software (St. Jude Medical Systems AB, Uppsala, Sweden) during all pacing configurations and during intrinsic rhythm. LVdP/dtmax at baseline and during the different pacing protocols was calculated over an interval of 15 s; premature ventricular contractions were eliminated electronically. A period of 30 s was allowed to elapse after any change in pacing settings or lead position to allow hemodynamic stabilization. To minimize the impact of respiration and physiological variation, each 15-s LVdP/dtmax value was measured during three separate recordings for each test configuration [15].

**Fig. 1:**
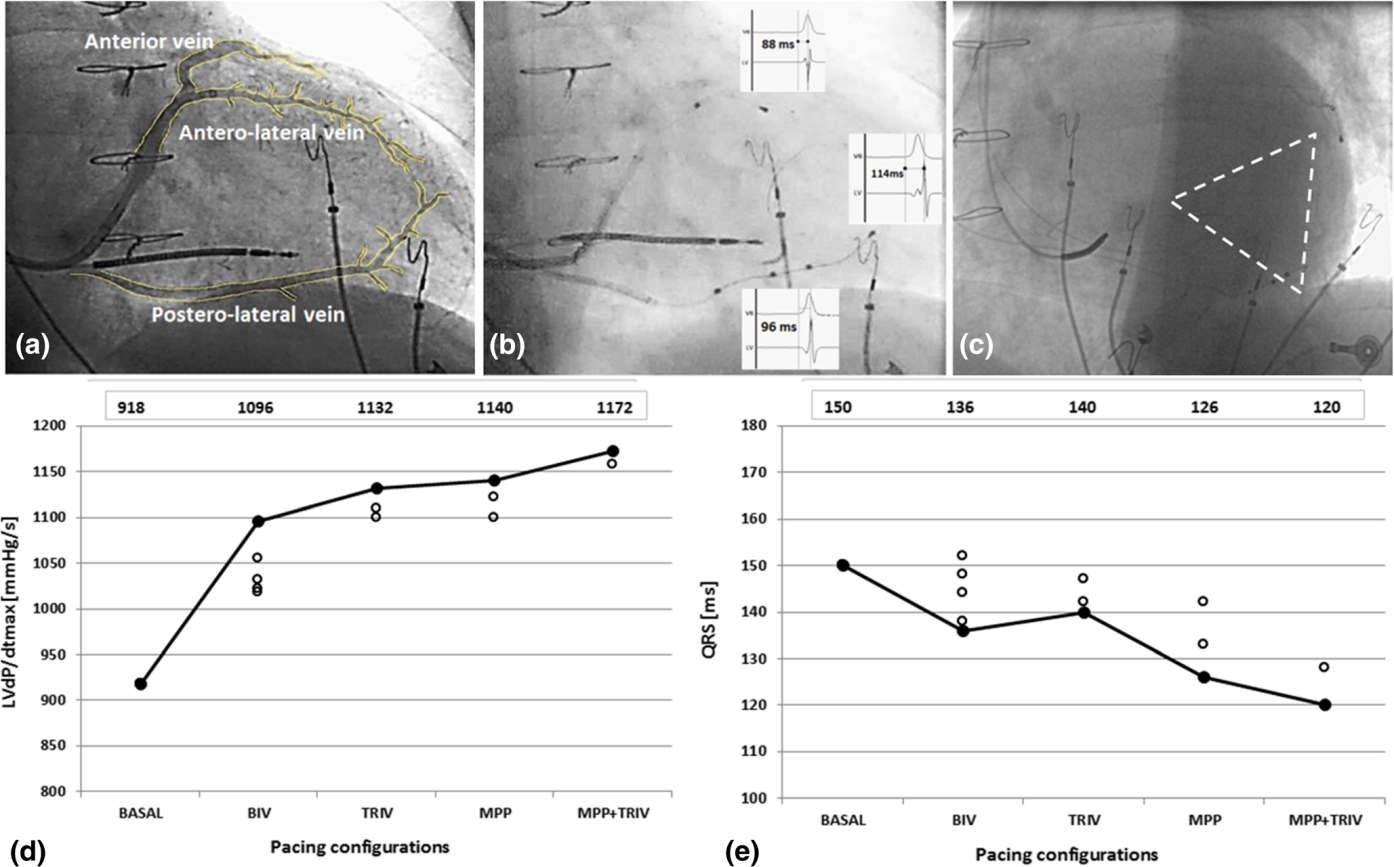
Example of the measurements taken on each patient—this patient was a 73-year-old male with permanent AF, NYHA class III, LBBB, ischemic CMP, and basal QRS 150 ms. **a** Coronary sinus venous angiography in RAO 20° showing an anterior vein, an antero-lateral vein, and a postero-lateral vein. **b** Final lead positions; the quadripolar lead is positioned in a postero-lateral vein and the bipolar lead is positioned in an antero-lateral vein. Q-LV measurements at each site are reported. **c** Final lead positions in LAO view. **d** Effects on LVdp/dtmax and on QRS width (**e**) during different pacing protocols. The values at the top of panels **d** and **e** indicate the best measurements. The dots in the graphs represent all the data collected for each pacing configuration. Abbreviations are in the text

In accordance with the guidelines, before CRT implantation, all patients underwent optimization of medical therapy, which included the up-titration of beta-blocker treatment. At the time of implantation, the mean heart rate of the whole population was 59 ± 7 (range 52–71 bpm). With the intention to obtain 100% of ventricular capture, the pacing protocol was set to 10 beats above the basal heart rate (mean pacing rate was 70 ± 4, range 65–80). Twelve-lead QRS morphology was examined beat by beat in the EP system in every pacing setting by an expert electrophysiologist to confirm continuous capture and avoid fusion beats. The first step was to place the quadripolar lead (Quartet™ 1458Q, St. Jude Medical) in every single vein available for cannulation. As per protocol, in every vein we collected data on the Q-LV interval measured from the proximal dipole, defined as LV1 (electrodes M3-P4, inter-dipole length 17 mm), and from the distal dipole, defined as LV2 (electrodes D1-M2 inter-dipole length 20 mm); we also assessed the hemodynamic effect by measuring the LVdp/dtmax at every site. A second bipolar lead, defined as LV3 (Quickflex Micro^TM^ 1258T, St. Jude Medical), was then implanted, usually in an anterior CS vein, and all the measurements were repeated in order to obtain all the possible combinations of pacing between the two leads. We started with BIV pacing with the proximal or distal dipole of the quadripolar lead (RV + LV1 or RV +LV2, depending on the best Q-LV), then TRIV (RV + LV1 + LV3 or RV + LV2 + LV3, depending on the best Q-LV), then MPP (RV + LV1 + LV2) and finally TRIV plus MPP (RV + LV1 + LV2 + LV3) in every vein (an explanatory example of all the data collected is displayed in Fig. 1). To explain our procedure more clearly, if patients had only two available veins, the quadripolar lead was implanted in the one with the longest Q-LV; if 3 or more veins were available, the quadripolar lead was tested in all veins, including those with non-optimal Q-LV. We did not collect data on veins that were visualized but not cannulated. MPP stimulation was performed by means of simultaneous pacing from the two dipoles of the quadripolar lead.

The sequence of site testing was not randomized in order to reduce as much as possible the procedural time.

**Fig. 2:**
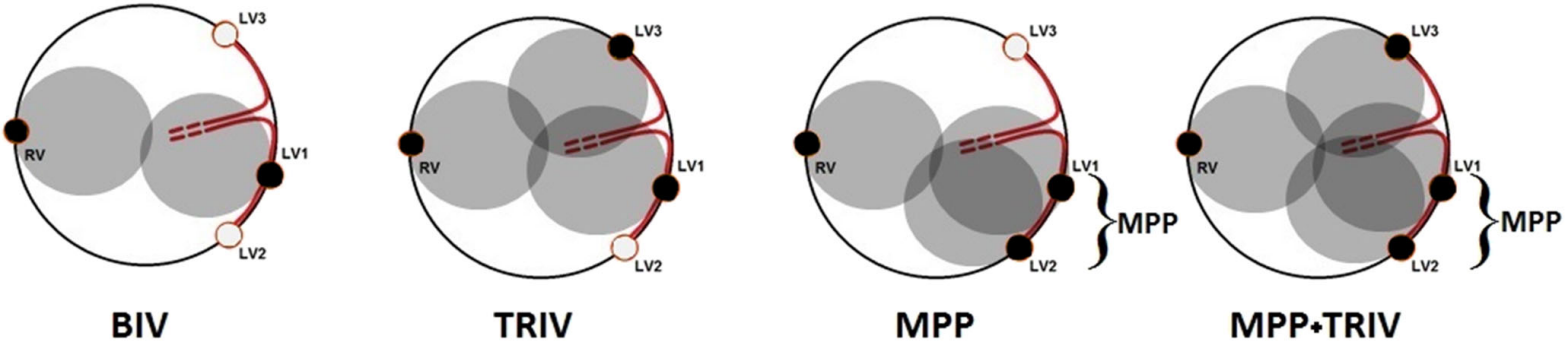
Pacing configurations (BIV = RV ± LV1 or BIV = RV ± LV2 depending on the best Q-LV; TRIV = RV ± LV1 ± LV3 or TRIV = RV ± LV2 ± LV3 depending on the best Q-LV; MPP = RV + LV1 + LV2; MPP + TRIV = RV + LV1 + LV2 + LV3)—black dots represent active dipoles, light gray dots represent passive dipoles, and gray circles suggest activation from active dipoles. BIV biventricular pacing (RV pacing plus LV pacing from one of the two dipoles of the quadripolar lead, LV1), TRIV tri-ventricular pacing (RV pacing plus LV1 plus a second LV bipolar pacing lead, LV3), MPP multipoint pacing (RV pacing plus pacing from both dipoles of the quadripolar lead, LV1 and LV2), MPP + TRIV tri-ventricular plus multipoint pacing (pacing from RV, LV1, LV2, and LV3)

The definitive position of the quadripolar lead was the optimal pacing site, defined as the most delayed site in terms of Q-LV, while the second bipolar lead was positioned in a different CS vein, usually in the most anatomically remote vein from the quadripolar lead. We evaluated the effect of the different pacing configurations on hemodynamics and QRS duration, as schematically illustrated in Table 1 and Fig. 2.

**Table 1:** Pacing configurations

	BIV	TRIV	MPP	MPP + TRIV
RV LEAD	Yes	Yes	Yes	Yes
LV1 LEAD (quadripolar) pacing from one of the 2 dipoles	Yes	Yes	Yes	Yes
LV2 LEAD (quadripolar) pacing from the other dipole	No	No	Yes	Yes
LV3 LEAD (bipolar)	No	Yes	No	Yes

*BIV* biventricular pacing (RV pacing plus LV pacing from one of the two dipoles of the quadripolar lead, LV1), *TRIV* tri-ventricular pacing (RV pacing plus LV1 plus a second LV bipolar pacing lead, LV3), *MPP* multipoint pacing (RV pacing plus pacing from both dipoles of the quadripolar lead, LV1 and LV2), *MPP + TRIV* tri-ventricular plus multipoint pacing (pacing from RV, LV1, LV2, and LV3)

In all configurations, pacing was performed by means of a triple-chamber pacing system analyzer (Merlin EX3100 PSA, St. Jude Medical). TRIV was obtained by connecting the bipolar LV lead to the atrial channel of the PSA, and by programming the atrio-ventricular delay to the minimum available value of 25 ms. The RV-LV delay was always set at 0 ms. During MPP, the LV1 and LV2 of the quadripolar lead were paced simultaneously from the LV output of the pacing system analyzer by means of a custom-made epsilon-shaped adapter. TRIV+MPP was obtained by combining the previous configurations.

The two LV leads were implanted definitively, as per protocol in our center. The bipolar LV lead was connected to the atrial port of the device and programmed with the shortest available AV delay (25 ms).

### Statistical analysis

Our aim was to evaluate the improvement in ventricular function, as estimated by the change in LV-dP/dtmax and QRS-width from the basal measurement, obtained by switching the cardiac pacing protocol from single-site BIV, TRIV, MPP to MPP + TRIV pacing in the same subject in a one-arm (intra-patient) study. The instrumental data collected were tabulated along with topographic and protocol information and patients’ clinical characteristics.

We used the “repeated measures analysis of variance” to estimate variations in within-subject measurements of LVdp/dtmax and QRS-width; the Greenhouse-Geisser adjustment was applied to degrees of freedom, and equal weights were attributed to measurements. The Bonferroni correction was used for pairwise planned comparisons between the pacing protocols.

The analyses were conducted on “all measurements” collected from patients (78 measurements).

## Results

The implantation of two LV leads was successfully performed in all patients. No procedure-related complications were reported. The characteristics of the patient population are shown in Table 2.

**Table 2:** Patient characteristics

Variable	
Total population (*n*)	15
ICM (*n*; %)	7; 46.7%
Age (years)	76 ± 7
Male (*n*; %)	13; 86.7%
NYHA class (*n*; %)	
II	1; 6.7%
III	13; 86.7%
IV	1; 6.7%
Atrial fibrillation (*n*; %)	15; 100%
LVEF (%)	33 ± 7%
ESVi (ml/m^2^)	65.7 ± 20.2
MR degree (*n*; %)	
1	5; 33%
2	4; 27%
3	3; 20%
4	3; 20%
QRS (ms)	178 ± 25
LBBB (*n*; %)	9; 60.0%
RBBB (*n*; %)	3; 20.0%
IVCD (*n*; %)	2; 13.3%
PM DEP (*n*; %)	1; 6.7%
CRT-P/CRT-D (*n*; %)	7; 46.7%/8; 53.3%
NTproBNP (pg/ml)	8.508 ± 5.124
Medication use (*n*; %)	
β-Blockers	11; 73%
ACE-ARB	7; 47%

*ICM* ischemic cardiomyopathy, *NYHA* New York Heart Association, *LVEF* left ventricular ejection fraction, *ESVi* End Systolic Volume index, *LBBB* left bundle branch block, *RBBB* right bundle branch block, *IVCD* inter-ventricular conduction delay, *PM DEP* pacemaker dependent, *CRT-P* CRT-pacemaker, *CRT-D* CRT-defibrillator, *NT-proBNP* N-terminal pro b-type natriuretic peptide, *ACE-ARB* ACE inhibitors (angiotensin converting enzyme inhibitors) and ARB (angiotensin-receptor blockers)

The mean procedural time was 174 ± 27 min, and the total fluoroscopy time was 36 ± 8 min. On average, 2.7 ± 0.7 veins per patient were cannulated and 5.2 ± 1.9 pacing sites were evaluated.

The final positions of the LV pacing leads are reported in Table 3.

**Table 3:** Final lead positions and Q-LV measurements for quadripolar and bipolar leads in each patient

Pt no.	Lead 1 (quadripolar)	Q-LV Quad (ms)	Lead 2 (bipolar)	Q-LV Bip (ms)	Explored veins/sites tested
1	Mid-basal posterior	122	Basal lateral	68	3/7
2	Mid-basal lateral	100	Mid-anterior	66	3/7
3	Mid-basal lateral	132	Mid-posterior	104	3/8
4	Mid-basal lateral	114	Mid-antero-lateral	86	2/4
5	Mid-apical postero-lateral	152	Mid-anterior	74	3/6
6	Mid-basal lateral	94	Basal anterior	52	2/3
7	Mid-basal lateral	124	Basal anterior	68	3/7
8	Mid-apical lateral	110	Basal anterior	70	3/7
9	Mid-basal lateral	166	Mid anterior	156	2/3
10	Mid-basal lateral	108	Mid antero-lateral	66	2/3
11	Mid-basal postero-lateral	114	Basal antero-lateral	88	4/6
12	Mid-basal postero-lateral	92	Basal anterior	62	4/7
13	Mid-basal lateral	134	Mid-postero-lateral	102	2/3
14	Mid-basal postero-lateral	150	Mid-anterior	86	2/3
15	Mid-basal postero-lateral	116	Basal anterior	22	3/4

Quad lead position means the area covered by the entire quadripolar complex. Total numbers of veins and explored sites in each patient are also displayed

**Fig. 3:**
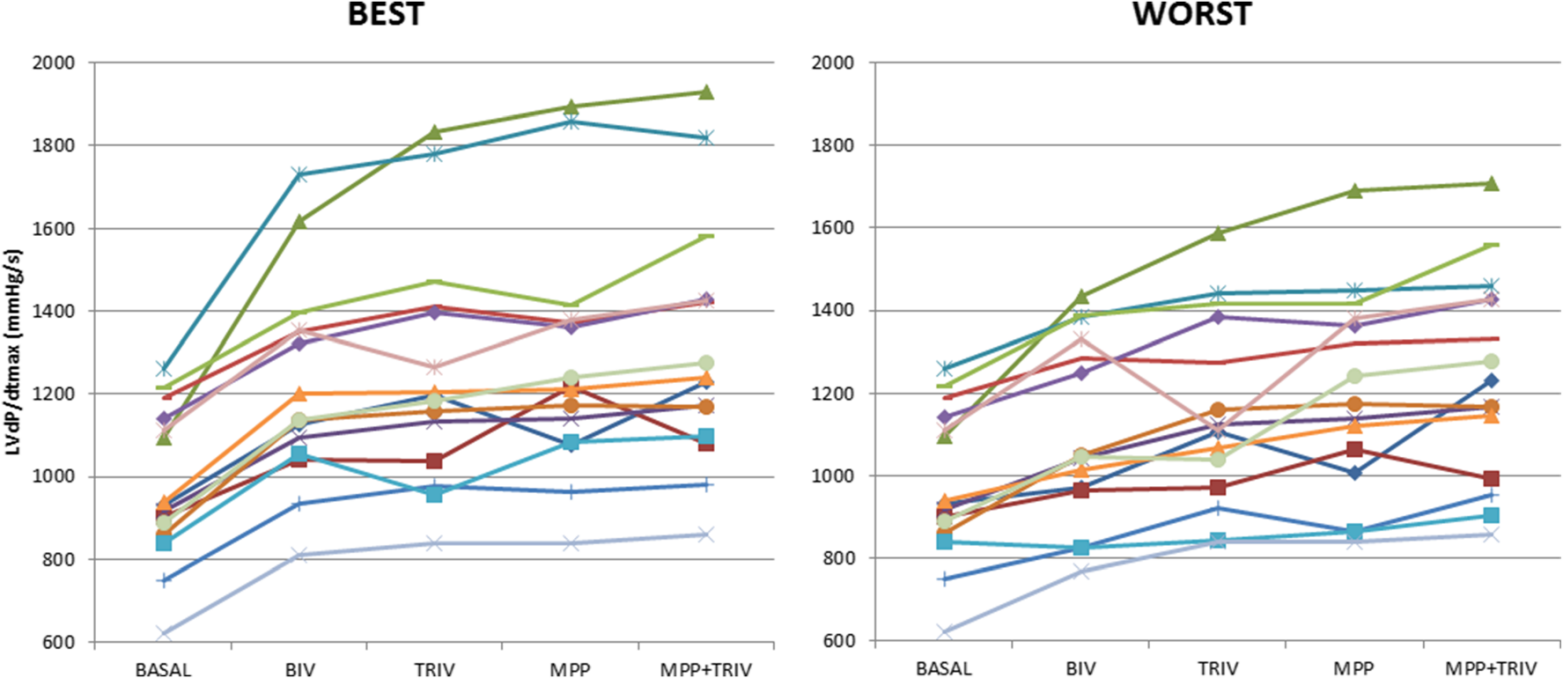
Intra-patient gain in LVdp/dtmax of each of the 15 patients in the cases of the best and worst measurements for every pacing modality

**Fig. 4:**
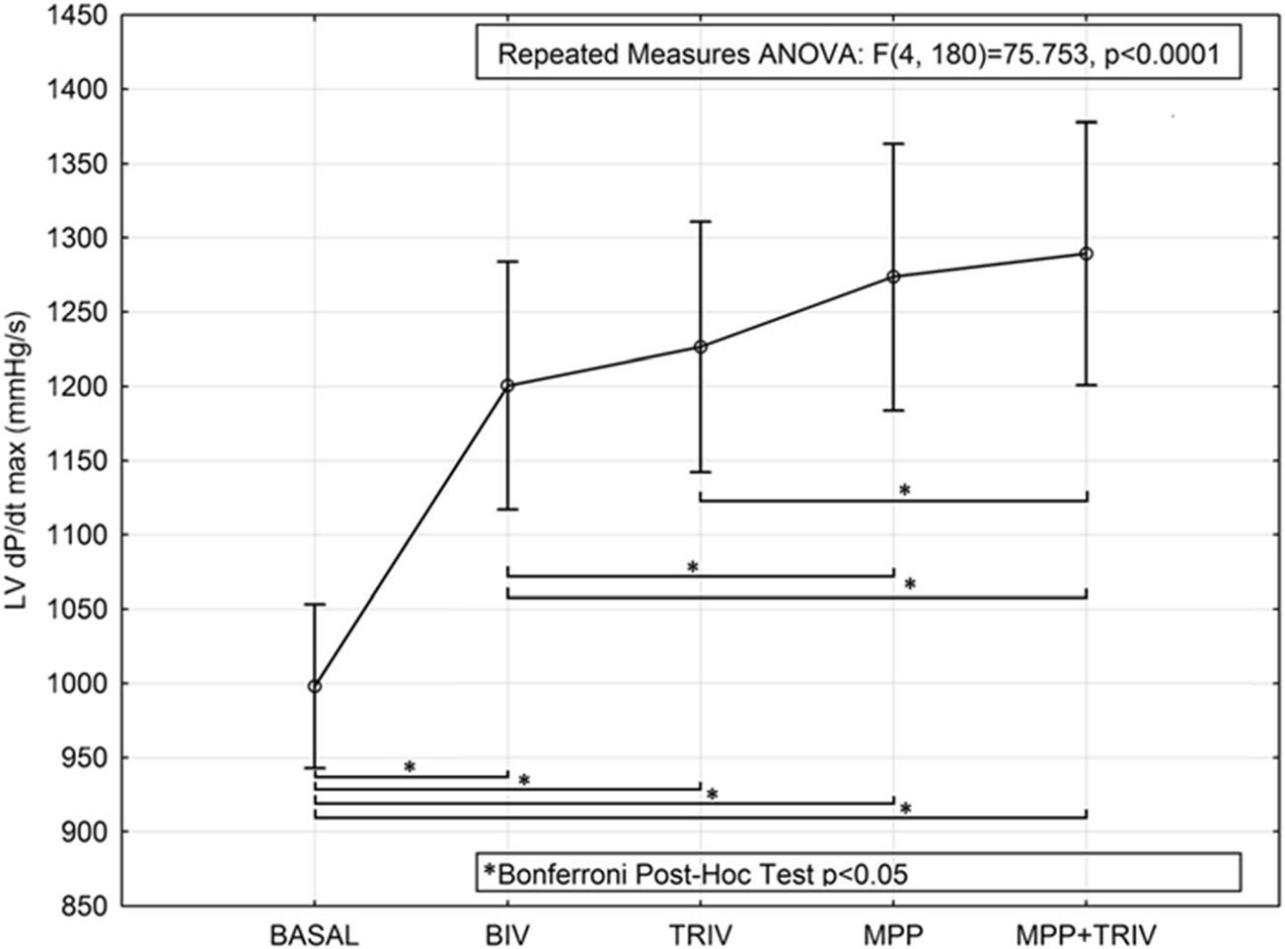
Gain in LVdp/dtmax yielded by the different pacing modes, during biventricular (BIV), triple ventricular (TRIV), multipoint (MPP), and MPP + TRIV pacing protocols vs baseline (considering all pacing sites, 78 series of measurements in the 15 patients)—Bonferroni post-hoc analysis showed significant differences from basal in all pacing configurations; significant differences were observed between BIV and MPP, between BIV and MPP + TRIV, and between TRIV and MPP + TRIV

Conventional BIV pacing resulted in a 202-mmHg/s increase in LVdp/dtmax (from 998 ± 186 to 1200 ± 281 mmHg/s). TRIV pacing caused a small, non-significant, further increase to 1226 ± 284 mmHg/s, whereas MPP and MPP + TRIV increased LVdp/dtmax significantly above the BIV pacing level (to 1274 ± 303 and 1289 ± 298 mmHg/s, respectively; repeated measures ANOVA; *p* < 0.001 followed by Bonferroni post-hoc analysis). Figure 3 displays the LVdp/dtmax intra-patient response of each of the 15 patients in the cases of the best and worst measurements for every pacing modality. Figure 4 displays the gain in within-subject measurements.

**Fig. 5:**
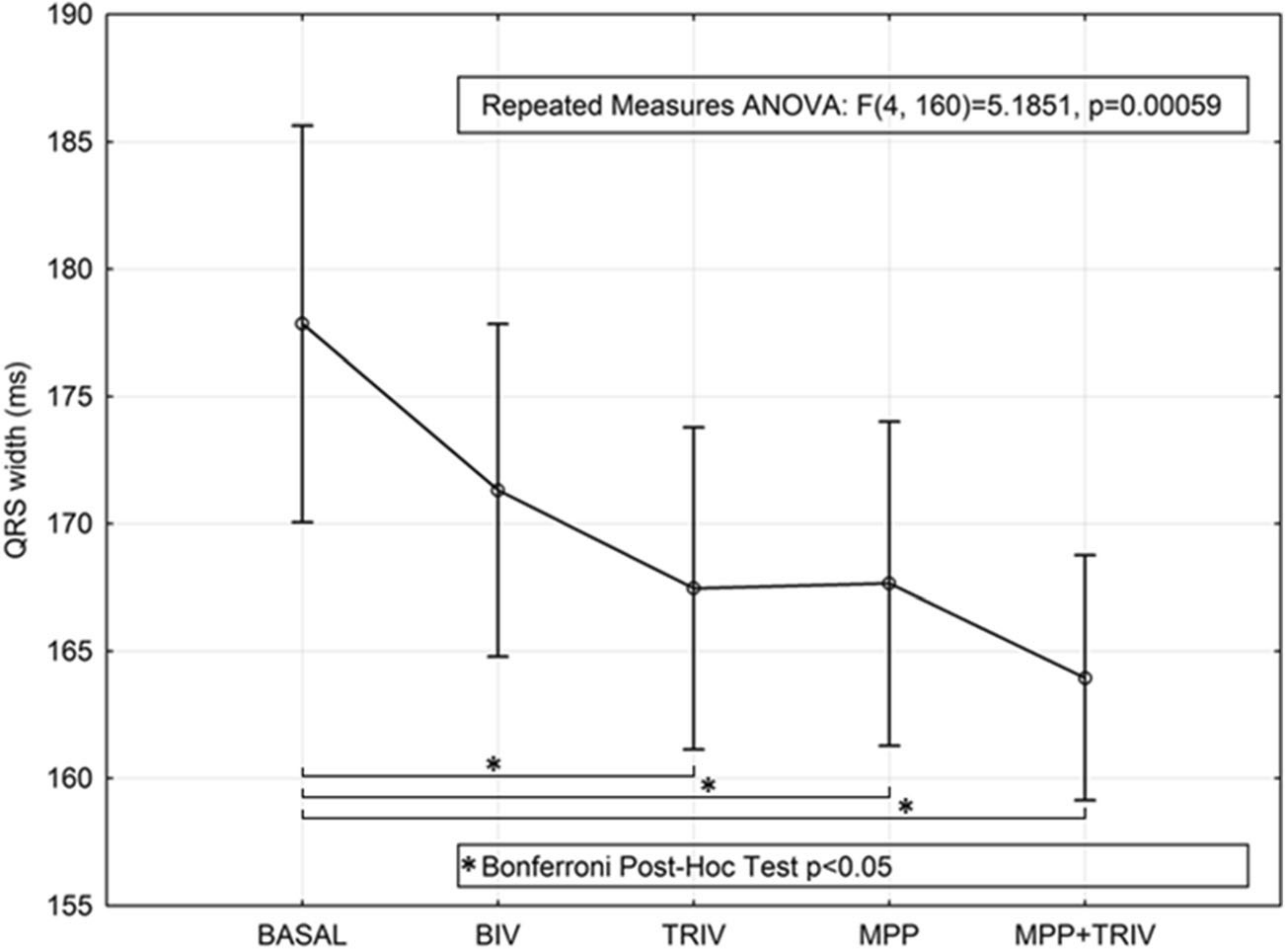
Effects on QRS width during biventricular (BIV), triple ventricular (TRIV) by means of two LV leads, multipoint (MPP), and multipoint plus second LV lead (MPP-TRIV) pacing protocols vs baseline (considering all pacing sites, 78 measurements)—Bonferroni post-hoc analysis showed significant differences from the baseline in all pacing configurations

The effects of the different pacing protocols on QRS width are reported in Fig. 5. TRIV, MPP, and their combination reduced QRS duration significantly in comparison with the baseline.

Percentage increase in LVdp/dtmax and percentage reduction in QRS width during the different pacing protocols are summarized in Table 4.

**Table 4:** Percentage changes in LVdp/dtmax (repeated measures ANOVA; *p* < 0.0001) and in QRS width (repeated measures ANOVA; *p* < 0.0001)

	Basal	BIV	TRIV	MPP	MPP + TRIV	*p*
LVdp/dtmax	998 mmHg/s	+ 20%	+ 23%	+ 28%	+ 29%	< 0.01
QRS	177 ms	− 3%	− 6%	− 6%	− 9%	< 0.01

*p* values for difference combinations related to gain in LVdP/dtmax and QRS width during pacing protocols are reported in Tables 5 and 6, respectively.

**Table 5:** Gain in LVdP/dtmax during different pacing modes—*p* values for difference combinations

dP_dt_BASAL	–				
dP_dt_BIV	0.0000	–			
dP_dt_TRIV	0.0000	1.0000	–		
dP_dt_MPP	0.0000	0.0017	0.1394	–	
dP_dt_MPP+TRIV	0.0000	0.0001	0.0114	1.0000	–
	dP_dt_BASAL	dP_dt_BIV	dP_dt_TRIV	dP_dt_MPP	dP_dt_MPP+TRIV

**Table 6:** Effects on QRS width during different pacing modes—*p* values for difference combinations

QRS_BASAL	–				
QRS_BIV	0.4772	–			
QRS_TRIV	0.0182	1.0000	–		
QRS_MPP	0.0220	1.0000	1.0000	–	
QRS_MPP+TRIV	0.0004	0.2592	1.0000	1.0000	–
	QRS_BASAL	QRS_BIV	QRS_TRIV	QRS_MPP	QRS_MPP+TRIV

## Discussion

Our data indicate that, in CRT patients with AF, pacing from a wider area in the LV acutely increases contractility more than standard BIV does. Most importantly, MPP increases contractility at least as much as TRIV, while it has the advantage of requiring only one LV lead. Combining MPP + TRIV can increase contractility even further, indicating that increasing the area of early activation on the LV increases the hemodynamic effect.

### Increasing the early activated area in the LV

The present study showed a two-step increase in the acute hemodynamic effect of CRT, beyond that due to conventional BIV pacing: a first increase yielded by TRIV or MPP alone and a further increase yielded by combining the two approaches. Because each of the two approaches aims to increase the size of the early activated region, these data support the idea that increasing this region is beneficial for resynchronization; this is also evidenced by a stepwise reduction in QRS duration. Several studies support the idea that QRS narrowing by CRT is a strong determinant of both echocardiographic and clinical response to CRT [16, 17].

While TRIV pacing can be considered to create an early activated region with a largely circumferential orientation (between two contributory venous zones), MPP creates a more baso-apically oriented zone. It is interesting that the orientation of the early activated zone does not seem to matter, as both approaches result in similar hemodynamic and electrophysiological benefit in comparison with conventional BIV pacing.

Finally, the observation that the combination of TRIV and MPP further improves CRT response is a further argument in favor of the idea that increasing the early activated region on the LV increases the effect of CRT. This may raise the question of what the optimal size of this early activated region may be. However, animal studies indicate that the additional hemodynamic benefit of pacing more than four sites may be small [18].

### Multisite vs. multipoint pacing

The idea of pacing from multiple sites has already been expounded upon by various authors [6, 7, 19], who used two leads in two different veins of the CS tree (TRIV). This technique has been demonstrated to be feasible and has yielded promising results from a clinical point of view, providing improvement in ejection fraction (EF) and ventricular remodeling [6]. Other authors have demonstrated a favorable effect of triple-site pacing on NYHA class in comparison with standard dual-site pacing. In a study by Rogers et al. [20], triple-site pacing was obtained by applying dual-site pacing to either the right or left ventricle and proved significantly superior to bipolar pacing in terms of the 6-min walking test, Minnesota living with HF score, ventricular remodeling, and EF.

In a study by Behar [21], 19 patients were paced at two different LV sites by means of two LV leads connected to a bifurcating adapter. While this technique yielded beneficial hemodynamic results on implantation, it also proved problematic in patients in sinus rhythm. Indeed, the two LV leads need to be connected to the device through an adapter and are stimulated from one source, while they may have different impedances. On the other hand, TRIV pacing in patients with AF, as in the study by Leclercq [6] and in our study, is more feasible because the second LV lead is connected to the atrial port. However, this solution is limited by the minimum AV interval, which is between 25 and 30 ms in the majority of commercially available devices.

The introduction of quadripolar technology has enabled a larger area to be paced from a single multipolar lead, and initial experiences documented significant improvements in hemodynamic [8, 9] and mechanical dyssynchrony [10, 11]. Our previous experience [22] extended the concept of the benefit of MPP, in that acute hemodynamic improvement was obtained in every single vein; indeed, on studying 29 patients with an average of 3.2 veins and 6.3 pacing sites per patient, we observed an improvement in LVdp/dtmax at all sites, including the best and worst positions, which suggests that this pacing modality is widely applicable. Our acute results were confirmed in a subsequent study in which MPP, optimized on implantation as previously described, proved significantly superior in terms of remodeling and clinical status to conventional BIV without optimization [23]. Pappone also demonstrated the positive effects of MPP at 3-month and 1-year follow-up examinations [24, 25]. Finally, a large multicenter Italian experience confirmed the favorable effects of MPP in comparison with standard BIV [26]. Recently, the IDE study met the pre-specified hypothesis of non-inferiority of MPP to standard BIV in terms of safety and effectiveness. Moreover, among patients randomized to MPP, those paced from anatomically distant poles displayed a significantly higher rate of response than those paced from close poles [27].

The question of whether acute improvement in LVdP/dtmax predicts long-term clinical benefit needs more evidence. However, our previous experience [23] indicates that, on implantation, acute optimization by means of LVdp/dtmax and electrical delay correlates positively with clinical and remodeling improvement at one-year follow-up. Previously, Duckett found that a 10% increase in LVdp/dtmax on pacing predicted LV reverse remodeling at 6-month follow-up [28].

The present study provides the first head-to-head comparison between the two modalities of pacing a larger area, multisite (TRIV) and multipoint pacing (MPP). The finding that MPP yields at least the same hemodynamic benefit as TRIV pacing suggests that MPP may be preferred to TRIV pacing, as the implantation technique of MPP technology is as simple as that of a bipolar LV lead and avoids placement of a second lead, with its accompanying increased risk of dislodgement.

### Limitations

This was an acute, single-center, non-randomized study; the patient sample size was limited, and the pacing protocol configurations were conditioned by the limitations of the currently available technology. Moreover, as outlined in the methods section, due to the long protocol procedure and in order to reduce as much as possible the duration of the implant, we did not randomize the pacing protocol series. As result, the lack of a randomization of the protocol might have influenced the hemodynamics by introducing linear effects. Furthermore, the acute hemodynamic results may not lead to long-term clinical benefit. The implications of this study should be confirmed in a larger, randomized, prospective, multicenter, long-term study.

## Conclusions

Pacing from a wider area, as in TRIV and MPP, increases electrophysiological and hemodynamic benefits in comparison with standard BIV pacing, and a further improvement is achieved by TRIV + MPP. This suggests that a larger early activated region increases the efficacy of CRT. In accordance with the concept that “less is more,” it appears that MPP, which requires only one lead, is probably the most convenient technique in terms of risk/benefit ratio.
